# Chemopreventive Agent 3,3′-Diindolylmethane Inhibits MDM2 in Colorectal Cancer Cells

**DOI:** 10.3390/ijms21134642

**Published:** 2020-06-30

**Authors:** Xiang Gao, Jingwen Liu, Kwang Bog Cho, Samanthreddy Kedika, Bin Guo

**Affiliations:** Department of Pharmacology and Pharmaceutical Sciences, College of Pharmacy, University of Houston, Houston, TX 77204, USA; xgao23@central.uh.edu (X.G.); jliu79@central.uh.edu (J.L.); kcho7@central.uh.edu (K.B.C.); skedika@uh.edu (S.K.)

**Keywords:** 3,3′-Diindolylmethane, MDM2, colorectal cancer, p53, cancer prevention

## Abstract

3,3′-Diindolylmethane (DIM) is a naturally derived chemopreventive compound. It comes from glucobrassicin, an indole glucosinolate enriched in cruciferous vegetables, and is formed in the acidic environment of the stomach after ingestion. Mouse double minute 2 homolog (MDM2) is an important, multi-functional oncogenic protein and it has been well recognized for its negative regulation of the tumor suppressor protein p53. We discovered a novel mechanism of action of DIM, that it directly inhibits MDM2 in multiple colorectal cancer (CRC) cell lines. Treatment with DIM decreased MDM2 at messenger RNA (mRNA) and protein levels, inhibited cancer cell proliferation, and induced cell cycle arrest and apoptosis. DIM-induced decrease of MDM2 is p53-independent and is partly mediated by proteasome degradation of MDM2, as blocking of the proteasome activity reversed MDM2 protein inhibition. Overexpression of MDM2 blocked DIM’s effects in growth suppression and apoptosis induction. When combined with imidazoline MDM2 inhibitors (Nutlin-3a and Idasanutlin/RG-7388), synergism was observed in cancer cell growth inhibition. In summary, our data support a new mechanism of action for DIM in direct inhibition of MDM2. The identification of MDM2 as a novel DIM target may help develop a new strategy in CRC prevention.

## 1. Introduction

Colorectal cancer (CRC) is a gastrointestinal malignancy that combines both colon and rectal cancers because of the shared clinical features. It is the third most common malignant disease globally, with an overall 5-year survival of 64%. The prognosis decreases to a 13% survival rate if diagnosis and intervention are delayed [1]. Chemotherapy and targeted therapy are included in the current therapeutic guidelines. While chemotherapy drugs kill rapidly dividing cells and exhibit high toxicities, monoclonal antibodies and tyrosine kinase inhibitors (TKIs) are designed to specifically target receptors on cancer cells, such as the epidermal growth factor receptor (EGFR) and several oncogenic receptors such as tyrosine kinase (RTK) [2,3,4]. Mutations of oncogenes and the occurrence of multi-drug resistance, as well as the intolerable side effects, limit the efficacy of treatment [5]. New strategies for CRC prevention and treatment are urgently needed.

The mouse double minute 2 homologue (*MDM2*) is an extensively studied oncogene which encodes the MDM2 protein, a major negative regulator of the p53 tumor suppressor. The inhibition of p53 function by MDM2 is achieved through at least three different mechanisms: (1) MDM2 binding to p53, (2) exporting p53 from the nucleus to the cytoplasm and blocking p53 function as a transcription factor, (3) inducing p53 ubiquitination and degradation [6]. MDM2 is an important target of cancer drug development. There are currently 18 ongoing clinical trials for MDM2 inhibitors in the US [7], including RG-7388 (idasanutlin), a third generation of *cis*-imidazoline MDM2 antagonist and a derivative from nutlin [8,9].

We have previously studied the epigenetic mechanism of action of 3,3′- Diindolylmethane (DIM) in colon cancer [10]. DIM is a natural chemopreventive compound that is formed from indol-3-cabinol (I3C) and has been reported to have various anti-cancer mechanisms in CRC [11,12,13,14,15,16]. DIM can be found in cruciferous vegetables such as broccoli, Brussels sprouts, cabbage and kale. Here, we report our new findings that DIM directly inhibits MDM2 in colorectal cancer cells. Our data suggest a novel mechanism of action of DIM in tumor suppression. Our results also show synergistic anti-cancer activity when DIM is combined with cis-imidazoline MDM2 inhibitors, a potential therapeutic strategy for CRC.

## 2. Results

### 2.1. DIM Inhibits MDM2 Protein in Colorectal Cancer Cells

**Figure 1:**
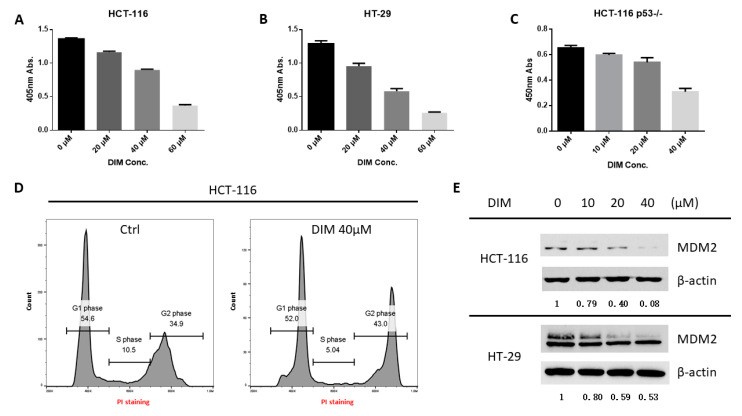
3,3′-Diindolylmethane (DIM) decreased MDM2 protein in colon cancer cells. (**A**) HCT-116, (**B**) HT-29 cells, and (**C**) HCT-116 p53 null cells were treated with various doses of DIM for 24 h. Cell proliferation was analyzed by WST-1 assay. (**D**) HCT-116 cells were treated with 40 μM DIM for 24 h then harvested and stained with Propidium iodide for cell cycle analysis. DIM induced cell cycle arrest in the G2 phase where the G2 population increased from 34.9% to 43.0%. (**E**) HCT-116 and HT-29 cells were treated with the indicated concentrations of DIM for 24 h. Cells were harvested, and the lysates were analyzed by Western blotting with the indicated antibodies. All experiments were repeated three times.

As a cancer preventive agent, DIM was able to inhibit the proliferation of colorectal cancer cell lines expressing wild-type or mutant p53 (Figure 1A-C), as well as other types of cancer cells (Figure S1A,B). In HCT-116 cells, DIM-induced growth inhibition was associated with G2 cell cycle arrest (Figure 1D). Interestingly, we found that DIM treatment significantly decreased the protein levels of MDM2 in both HCT-116 (p53 wild-type) and HT-29 (p53 mutant) CRC cells, in a dose-dependent manner (Figure 1E). In addition, DIM treatment decreased MDM2 in other cancer cell lines including A549 (p53 wild-type), NCI-H358 (p53 null), MCF-7 (p53 wild-type), and MIAPaCa-2 (p53 mutant) cells (Figure S1C), suggesting a common mechanism of action in various types of cancers. To eliminate any potential impact of antibody epitope masking, we used another clone of MDM2 (mAb SMP-14) which binds a different epitope from clone 2A10 and confirmed the inhibition of MDM2 (Figure S2).

### 2.2. DIM Induces Proteasome-Mediated MDM2 Degradation

**Figure 2:**
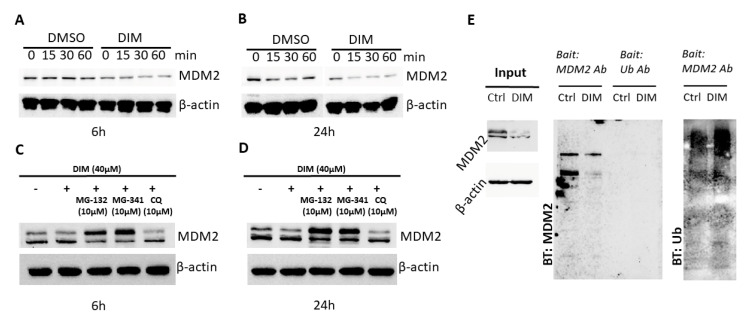
DIM induced proteasome-mediated degradation of MDM2 in HCT-116 cells. HCT-116 cells were pretreated with 40 μM DIM for (**A**) 6 h and (**B**) 24 h before cycloheximide (CHX) (at a final concentration of 10 μg/mL) was added into the culture. Cells were then lysed at the indicated time points after CHX application. (**C**,**D**) HCT-116 cells were treated with various proteasome or autophagy inhibitors. HCT-116 cells were incubated for (**C**) 6 h and (**D**) 24 h with 40 μM DIM and 10 μM of the indicated inhibitor. Cell lysates were analyzed by Western blotting with the indicated antibodies. (**E**) HCT-116 cells were treated with 40 μM DIM for 24 h. Co-immunoprecipitation was performed using an anti-MDM2 antibody, and the immunoprecipitates were blotted with anti-MDM2 or anti-ubiquitin antibodies.

**Figure 3:**
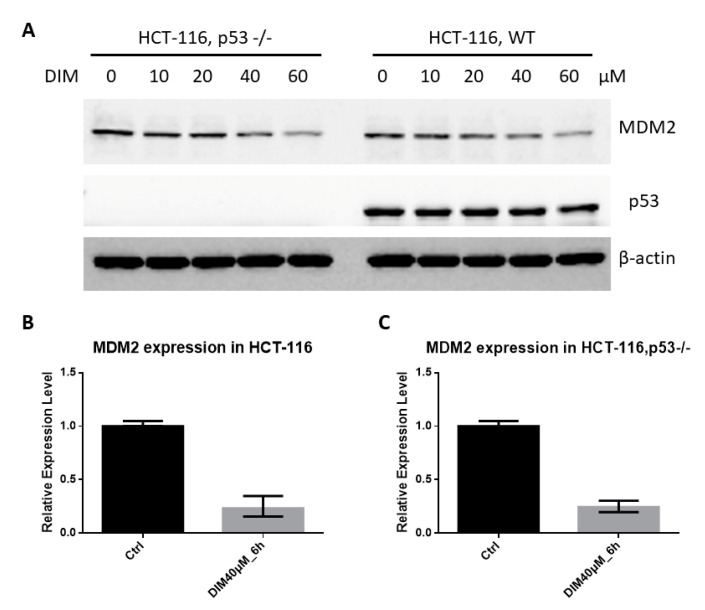
DIM inhibited MDM2 independent of p53. (**A**) Wild-type HCT-116 and p53 knockout HCT-116 cells were treated with various doses of DIM for 24 h. The cell lysates were analyzed by Western blotting with the indicated antibodies. (**B**) Wild-type HCT-116 cells and (**C**) p53 knockout HCT-116 cells were treated with 40 μM of DIM for 6 h. Real-time polymerase chain reaction (PCR) analysis of MDM2 gene expression was conducted as described in Materials and Methods.

To understand the mechanism of MDM2 inhibition, we treated cells with cycloheximide (CHX), which blocks protein synthesis in eukaryotic cells. CHX was added into culture media of HCT-116 cells that were pre-incubated with or without DIM. Time-lapsed sampling of MDM2 blotting showed an accelerated decrease of MDM2 protein in the presence of DIM (Figure 2A,B), indicating increased protein degradation after DIM treatment. To determine if the proteasome is involved in DIM-induced MDM2 degradation, we treated cells with the proteasome inhibitors MG-132 and MG-341. These proteasome inhibitors preserved MDM2 protein in the presence of DIM. In contrast, the autophagy inhibitor chloroquine (CQ) showed no effect on DIM-induced decrease of MDM2 (Figure 2C,D), indicating that autophagy is not a mechanism of MDM2 degradation during DIM treatment. MDM2 protein was notably elevated by these proteolytic inhibitors, i.e., MG-132 and MG-341 (Figure S3), but this increase was blocked by DIM. This is not surprising since DIM also inhibits MDM2 mRNA expression (see Figure 3 below). Using a co-immunoprecipitation experiment, we found increased MDM2 ubiquitination in cells treated with DIM (Figure 2E).

### 2.3. DIM Inhibition of MDM2 Is P53-Independent

The transactivation of MDM2 by p53 is an important feedback loop of the p53-MDM2 pathway [17,18]. To determine if p53 is involved in DIM-induced decrease of MDM2, we tested the effects of DIM in p53 negative HCT-116 cells. We found that DIM decreased MDM2 in p53 negative HCT-116 cells to a similar degree as that in p53 wild-type cells. Thus, DIM-induced downregulation of MDM2 is independent of p53 status in HCT-116 cells (Figure 3A). DIM decreased MDM2 in several cell lines carrying either wild-type or mutant p53 (Figure S1C), further supporting the p53-independent action of DIM. DIM treatment also decreased MDM2 mRNA expression. Real-time PCR analysis of MDM2 gene expression showed that DIM inhibited MDM2 mRNA expression independent of p53 status in HCT-116 cells (Figure 3B,C). We have previously shown that DIM induced p21 expression, G2 cell cycle arrest, and apoptosis in HT-29 cells [10]. Here, we found that DIM induced p21, p27 and apoptosis activator (PUMA) expression in HCT-116 cells (Figure S4). The activation of these cycle inhibitors (p21 and p27) and apoptosis activator (PUMA) may explain the p53-independent activity of DIM.

### 2.4. Prediction of DIM-MDM2 Interaction

**Figure 4:**
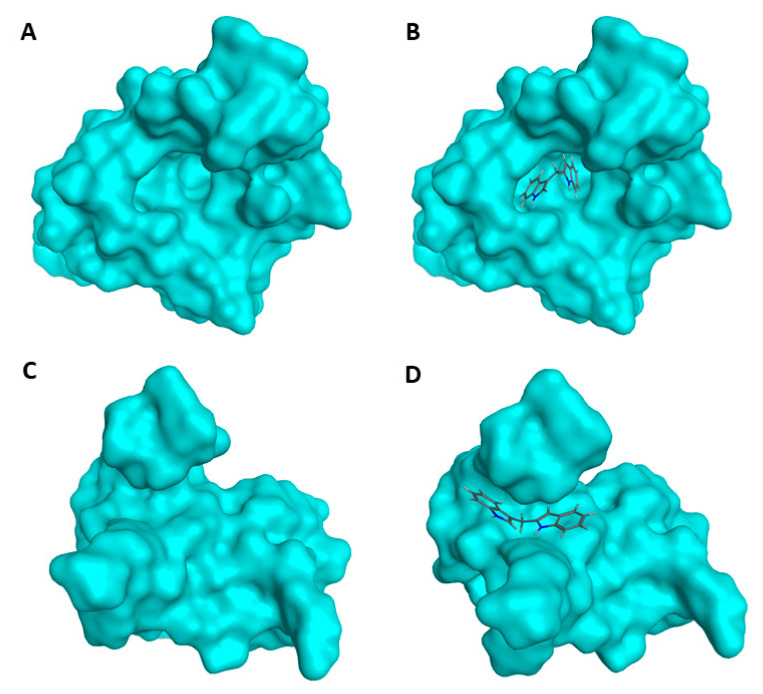
In-silico prediction of MDM2-DIM interaction. (**A**) N terminal structure (p53 binding site) of MDM2. (**B**) Molecular Operating Environment (MOE) software predicted interaction between DIM and the MDM2 N-terminal hydrophobic pocket. (**C**) C-terminal structure (Really Interesting New Gene (RING) domain) of MDM2. (**D**) MOE predicted the binding of DIM to the RING domain grove in MDM2.

As DIM induced MDM2 ubiquitination and degradation, we hypothesized that DIM may bind to MDM2 directly and trigger its degradation. We used Molecular Operating Environment (MOE) software to perform in-silico docking to see if there is a possible interaction between MDM2 and DIM. The prediction showed that DIM may occupy the hydrophobic pocket located in the N terminal of MDM2 (Figure 4A,B). In addition, there is another potential binding site in the C terminal, the Really Interesting New Gene (RING) domain of MDM2 protein, where DIM could also bind (Figure 4C,D).

### 2.5. MDM2 Inhibition Contributes to DIM’s Anti-Cancer Activity

**Figure 5:**
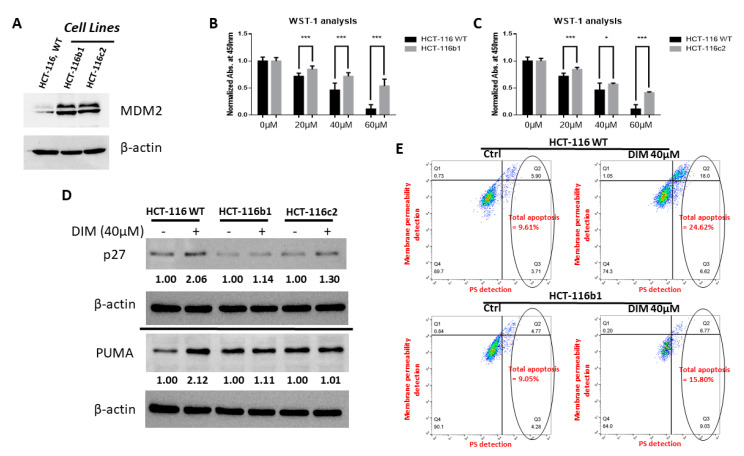
Overexpression of MDM2 reduced the anti-cancer activity of DIM. (**A**) MDM2 overexpressing clones of HCT-116 cells (HCT-116b1 and HCT-116c2) were created and MDM2 expression was confirmed by Western blotting. (**B**,**C**) Wild-type and MDM2 overexpressing clones of HCT-116 cells were treated with various doses of DIM for 24 h. DIM was added within 24 h of cell seeding to better compare the DIM effect on wild-type and MDM2 overexpression cells. Cell proliferation was assessed by WST-1 assay as described in Materials and Methods. Asterisk represents significance levels (analyzed by *t* tests) with *p* ≤ 0.05 (*), *p* ≤ 0.01 (**), and *p* ≤ 0.001 (***). All experiments were repeated three times; data shown are mean values + SD. (**D**) Western blotting showed that DIM induced smaller amounts of PUMA and p27 proteins in MDM2 overexpressing cells compared with HCT-116 wild-type cells. The amount of change of protein was noted in numbers compared with the corresponding control group. (**E**) Flow cytometry showed DIM induced a higher level of apoptosis in wild-type HCT-116 cells (total apoptosis population = 24.62%) compared with HCT-116b1 cells (total apoptosis population = 15.80%). Apoptosis was determined by phosphatidylserine (PS) staining with Apopxin™ dye. Necrosis as well as late stage apoptosis were determined by the loss of membrane integrity, detected using DNA Nuclear Green DCS1 dye.

To determine if the downregulation of MDM2 is important for the anti-cancer activity of DIM, we created MDM2 overexpression HCT-116 cells to investigate if exogenous expression of MDM2 would attenuate the anti-cancer activity of DIM. Two MDM2 overexpressing clones of HCT-116 cells (HCT-116b1 and HCT-116c2) were created (Figure 5A). When these MDM2 overexpressing clones and unmodified HCT-116 cells were treated with DIM, the cell lines overexpressing MDM2 showed resistance to DIM’s anti-proliferation as well as apoptosis-inducing activities (Figure 5B–E). Consistently, DIM treatment induced smaller amounts of p27 and PUMA in MDM2 overexpressing cells compared to the wild-type cells (Figure 5D). DIM was also able to decrease MDM2 level in MDM2 overexpressing cells (Figure S5). As DIM promotes proteasome-mediated MDM2 degradation, it is not surprising that exogenously expressed MDM2 can also be decreased by the treatment of DIM.

### 2.6. DIM Enhances the Anti-Cancer Activity of Cis-Imidazoline MDM2 Inhibitors

**Figure 6:**
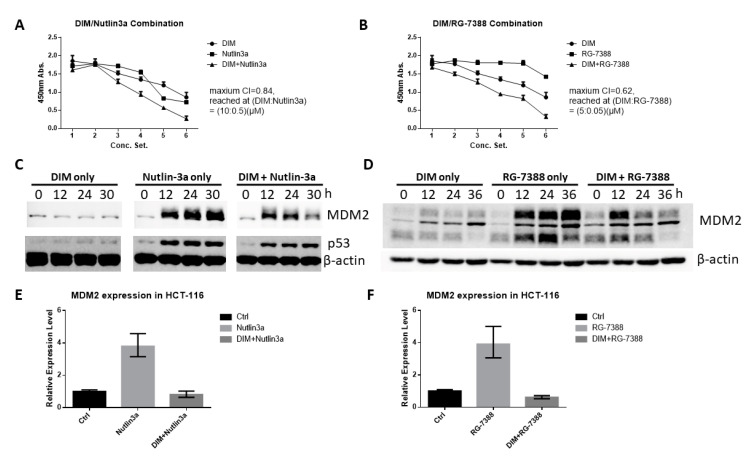
DIM enhanced the anti-cancer activity of Nutlin-3a and RG-7388. HCT-116 cells were treated with a single agent or DIM in combination with Nutlin-3a (**A**) or RG-7388 (**B**). Cell proliferation was determined by WST-1 assay. The combination index (CI) was calculated by CompuSyn [23]. (**C**) HCT-116 cells were treated with a single agent of Nutlin-3a (10 μM) or a combination of Nutlin-3a and DIM (40 μM). Western blotting was performed using the indicated antibodies. (**D**) HCT-116 cells were treated with a single agent of RG-7388 (5 μM) or a combination of RG-7388 and DIM (40 μM). Western blotting was performed using the indicated antibodies. (**E**,**F**) HCT-116 cells were treated for 6 h with a single agent or a combination of DIM (40 μM), Nutlin3a (10 μM), and RG-7388 (5 μM). MDM2 mRNA expression was analyzed as described in Materials and Methods.

To determine if DIM can enhance the anti-cancer activity of cis-imidazoline MDM2 antagonists, we treated HCT-116 cells with Nutlin-3a and RG-7388 alone or in combination with DIM, with the concentrations of the drugs shown in Table 1. The combination therapy of DIM with both antagonists showed stronger anti-proliferative effects than the single agent (Figure 6A,B). Treatment with Nutlin-3a or RG-7388 increased the levels of MDM2 protein (Figure 6C,D), possibly because the released p53 can upregulate MDM2 expression [19,20]. The increased MDM2 may protect cancer cells through p53-independent mechanisms [20,21,22]. However, combination with DIM prevented the Nutlin-3a and RG-7388-induced increase of MDM2 (Figure 6C,D), which may explain the synergistic effects in tumor suppression. Nutlin-3a and RG-7388 also increased MDM2 mRNA expression in HCT-116 cells, which was also blocked by DIM co-treatment (Figure 6E,F). The single agent or combination treatments have similar effects on p53 expression in HCT-116 cells (Figure 6C and Figure S6).

## 3. Discussion

The precursor of DIM, I3C, has been clinically used for recurrent respiratory papillomatosis (RRP) [24]. As a condensation product of I3C, DIM has been considered an important molecule that exerts I3C’s biological activities. In an animal model, DIM has a considerably longer half-life than I3C [25]. DIM can be detected after oral dosing in humans [26]. DIM has been investigated in several clinical studies for cancer prevention [27,28]. Various mechanisms of action have been studied to understand the role of DIM in cancer prevention [29,30,31]. Interestingly, inhibition of the ubiquitin E3 ligases has been shown to contribute towards I3C’s anti-cancer effect [32,33]. Thus, it is conceivable that the disruption of the ubiquitin-proteasome system in cancer cells by this class of pleiotropic phytochemicals may play a role in their anti-cancer activity. Our data suggest MDM2 as a novel target of DIM. This is significant because MDM2 is an important oncogene that plays a key role in the development and progression of various types of cancers [34]. Direct inhibition of DIM opens a new avenue for cancer prevention using bioactive compounds of food origin.

MDM2 is inhibited by DIM at both mRNA expression level and protein level through proteasome-mediated proteolysis. This mechanism is distinct from the well-studied MDM2 inhibitors such as Nutlin-3a and RG-7388, which mainly disrupt the interaction between MDM2 and p53. The anti-cancer activity of these cis-imidazoline MDM2 inhibitors thus relies on the presence of wild-type p53 [35,36]. In contrast, DIM decreases the levels of MDM2 protein and kills cancer cells without the need for wild-type p53. More importantly, as Nutlin-3a blocks the p53-MDM2 interaction, p53 is released from MDM2 and is able to bind to the MDM2 promoter and increase MDM2 expression (Figure 6C). The dramatically increased MDM2 protein may protect cancer cells from Nutlin-3a-induced apoptosis through various oncogenic mechanisms that have been linked to MDM2 [37,38]. Combination with DIM prevented this drawback from happening, as Nutlin-3a-induced increase of MDM2 is blocked by DIM (Figure 6C), and a similar result was found in DIM/RG-7388 combination. As a result, DIM can be used to enhance the therapeutic efficacy of MDM2 inhibitors such as AMG 232, which are currently in clinical trials [39,40].

The mechanism of how DIM induces MDM2 degradation is not known. An interesting hint may be the possible interaction between MDM2 and DIM at the RING domain (Figure 4D), which could interrupt the formation of the MDM2/mouse double minute X (MDMX) complex. As MDMX is capable of stabilizing the MDM2 protein, which would be otherwise subject to rapid self-degradation through self-ubiquitination [41,42], it is possible that the MDM2-DIM interaction may abolish the MDM2/MDMX complex and result in MDM2 degradation. Alternatively, DIM is known to bind to and activate the aryl hydrocarbon receptor (AhR) [43], which is an E3 ubiquitin ligase [44]. It is possible that DIM promotes MDM2 proteolysis through AhR-mediated activation of the proteasomal pathways.

We also found that DIM decreased MDM2 mRNA expression (Figure 3), which constitutes an additional mechanism of MDM2 inhibition. This action is apparently p53-independent, since similar degrees of inhibition were observed in the p53 knockout HCT-116 cells and the p53 wild-type cells. The MDM2 promoter is known to be regulated by p53 [43] and other transcription factors, including the thyroid hormone receptor [44] and the estrogen receptor ERα [45]. It is possible that DIM inhibits MDM2 mRNA expression by acting on these transcription factors. In this regard, DIM is known as a modulator of the estrogen receptor [46], suggesting a possible connection to the effects of DIM on MDM2 expression.

## 4. Materials and Methods

### 4.1. Cell Culture and Transfection

The cell lines HCT-116, LS-174T, HT-29, and HEK-293T were purchased from the American Type Culture Collection (ATCC). HCT-116 and LS-174T cells were cultured in a RPMI-1640 medium (Corning, Manassas, VA, USA). HT-29 and HEK-293T cells were cultured in Dulbecco’s modified Eagle medium (DMEM, Corning, Manassas, VA, USA). Both types of medium include 10% fetal bovine serum (Corning, Woodland, CA, USA). Penicillin-streptomycin solution (Corning, Manassas, VA, USA) was applied to all culture mediums to reach a final concentration of 100IU/100µg/mL before use. The pLenti-GIII-CMV-MDM2 plasmid (Applied Biological Materials, Richmond, BC, Canada) was used to transfect HCT-116 cells to create MDM2 overexpression clones, HCT-116b1 and HCT-116c2.

### 4.2. Drugs and Chemicals

DIM (cat. # D3232, LKT Laboratories, St. Paul, MN, USA), RG-7388 (cat. # 21532, Cayman, Ann Arbor, MI, USA), and Nutlin-3a (cat. # 44151, Millipore, Billerica, MA, USA) were dissolved/diluted in dimethyl sulfoxide (DMSO, Corning, Manassas, VA, USA) and applied to the cell culture to reach the indicated concentrations.

### 4.3. WST-1 Assay

For proliferation assays, WST-1 reagent (Takara, Mountain View, CA, USA) was applied to the cell culture. Absorbance at 450 nm of the medium from each sample was subsequently measured by an EMax™ Plus microplate reader (Molecular Devices, San Jose, CA, USA). Combination index (CI) in the combination study was calculated by CompuSyn (CompuSyn software, Version 1.0, Combosyn Inc., Paramus, NJ, USA).

### 4.4. Flow Cytometry

Cell cycle analysis was performed by PI (cat. # P1304MP, Invitrogen, Eugene, OR, USA) staining and cell death was analyzed by flow cytometry using the Apoptosis/Necrosis Assay Kit (cat. # ab176750, Abcam, Cambridge, MA, USA). Briefly, the cell culture with desirable confluence was treated by DIM for 24 h and then prepared into single cell suspension using a Cell Dissociation Buffer (cat. # 13151014, Gibco, Carlsbad, CA, USA). The subsequent staining followed the manufacturer’s protocols. Detection was performed on an Accuri™ C6 (BD Biosciences, Franklin Lakes, NJ, USA). Data analysis was conducted using FlowJo™ v10 software (Version 10.5.2, FlowJo. LLC, Ashland, OR, USA).

### 4.5. Blocking of Protein Synthesis and Degradation

For biosynthesis blocking assays, cells were incubated with DIM for the indicated length of time before CHX (cat. # 239764, Millipore, Billerica, MA, USA) was applied into the culture at a final concentration of 10 μg/mL. Cultures were then harvested and lysed in a time-lapse manner for further Western blot analysis. For proteolytic/autophagy blocking, MG-132 (cat. # C2211, Sigma, St. Louis, MO, USA), MG-341 (cat. # 10008822, Cayman, Ann Arbor, MI, USA), and CQ (cat. #14194, Cayman, Ann Arbor, MI, USA) were added together with DIM into cell cultures for incubation.

### 4.6. Western Blotting

Cells were lysed in RIPA lysis buffer (cat. # 97063-270, VWR, Solon, OH, USA). A cOmplete™ Protease Inhibitor Cocktail (cat. # 11836145001, Roche, Mannheim, Germany) was added to the lysis buffer before use. Protein concentration was determined by Bio-Rad DC protein assay (Bio-Rad, Hercules, CA, USA). After heating with a beta-mercaptoethanol (BME, Sigma, St. Louis, MO, USA) mixed Laemmli loading buffer (Bio-Rad, Hercules, CA, USA), protein samples were subjected to sodium dodecyl sulfate polyacrylamide gel electrophoresis (SDS-PAGE) and transferred to a nitrocellulose membrane. The membrane was blocked in 5% nonfat milk in Tris-buffered saline (TBS) for 1 h at room temperature and incubated overnight with primary antibodies (MDM2 monoclonal antibody, mAb, clone 2A10, cat. # OP-115 and cat. # MABE281, Millipore, Billerica, MA, USA; MDM2 mAb clone SMP-14, cat. # sc-965 Santa Cruz Biotechnology; p53 mAb clone DO-1, cat. # sc-126, Santa Cruz Biotechnology, Santa Cruz, CA, USA) at 4 °C and subsequently with appropriate secondary antibodies (Goat-anti-mouse polyclonal antibody, pAb, with horseradish peroxidase, HRP, conjugation, cat. # sc-2060, Santa Cruz Biotechnology, Santa Cruz, CA, USA). β-actin antibody (cat. # A3854, Sigma, St. Louis, MO, USA) was used for blotting reference. Blotting signals were developed with Pierce™ ECL reagents (Thermo Fisher) and exposure in a ChemiDoc^TM^ Touch imaging system (Bio-Rad, Hercules, CA, USA). The protein amount change was analyzed using ImageLab^TM^ software (Bio-Rad, Version 5.2.1, Hercules, CA, USA).

### 4.7. Co-Immunoprecipitation (Co-IP)

Cells were harvested and processed using a Pierce™ Co-Immunoprecipitation Kit (Thermo Fisher) following the manufacturer’s protocol. After the loading of column elute samples, SDS-PAGE, antibody incubation, and blotting developing were described in the Western blot. The ubiquitin (Ub) mouse mAb (cat. # sc-8017, Santa Cruz Biotechnology, Santa Cruz, CA, USA) and MDM2 mAb (cat. # MABE281, Millipore, Billerica, MA, USA) were used.

### 4.8. Real-Time PCR

Total RNA was isolated from cells using a RNeasy Micro Kit (Qiagen, Germantown, MD, USA). An amount of 40 ng total RNA was loaded into a reaction system containing M-MLV RT RNase (cat. # M368A), 5x RT buffer (cat. # M531A), a rRNasin^TM^ ribonuclease inhibitor (cat. # N251A), a PCR nucleotide mix (cat. # U144B) from Promega (Promega, Madison, WI, USA), and a random hexamer (cat. # 100026484) from Invitrogen (Life technologies, Carlsbad, CA, USA) for the reverse transcription reaction. The cDNAs were then used as templates to perform real-time PCR on a QuantStudio 3 Real-time PCR System (Applied Biosystems, Thermo Fisher Scientific Inc., Waltham, MA, USA), following the manufacturer’s protocol. The MDM2 gene expression was measured by real-time PCR using a TaqMan^TM^ gene expression assay (cat. # 4331182-Hs01066931_m1 for MDM2, cat. # 4331182-Hs99999901_s1 for 18s, Applied Biosystems, Foster City, CA, USA). Relative MDM2 expression levels were derived using a 18S RNA expression profile as the endogenous control. Error bars in control groups represent the SD within that group, whereas error bars for the treatment groups represent 95%CI which was calculated from below equations (Equations (1) and (2)):

(1) $$95\%\mathrm{CI}=2^{\left[\Delta \Delta CT\;\pm \;\sigma \left(\Delta \Delta CT\right)\right]}$$

(2) $$\sigma \left(\Delta \Delta CT\right)={\left(\left(\sigma {\left[\Delta CT_{control}\right]}^{2}\right)+\left(\sigma {\left[\Delta CT_{treatment}\right]}^{2}\right)\right)}^{\frac{1}{2}}$$

### 4.9. Molecular Modeling

The docking studies were performed using Molecular Operating Environment (MOE, Version 2018.01, Chemical Computing Group ULC., Montreal, QC, Canada) software [45]. Two different crystal structures of MDM2 (2VJE and 3LBK) were utilized for docking. Among these two complexes, only the MDM2 protein structure was further utilized for the docking analysis. The structures were first prepared using a “quick prep” tool to correct crystallographic defects and to perform energy minimization. Next, the binding pockets were identified using a “site finder” tool. Atom types were then reassigned to a MMFF94x (default- Amber10: EHT) force field and further used for docking with the DIM (energy minimized structure). Finally, the top predicted binding pose (that represents lower free binding energy or ΔG), corresponding to each complex was visualized.

## Figures and Tables

**Table 1:** Combination of DIM and MDM2 antagonists.

Concentration Setting #	1	2	3	4	5	6	Unit
DIM	0	5	10	20	30	40	μM
Nutlin-3a	0	0.1	0.5	1	5	10	μM
RG-7388	0	0.05	0.1	0.5	1	5	μM

## Supplementary Materials

Supplementary materials can be found at https://www.mdpi.com/1422-0067/21/13/4642/s1.

## Abbreviations

DIM	3,3′- Diindolylmethane
MDM2	Mouse double minute 2 homolog
MDMX	Mouse double minute X
I3C	Indol-3-cabinol
CRC	Colorectal cancer
mRNA	Messenger RNA
PCR	Polymerase chain reaction
TKI	Tyrosine kinase inhibitors
EGFR	Epidermal growth factor receptor
RTK	Receptor tyrosine kinase
CHX	Cycloheximide
PI	Propidium iodide
PS	Phosphatidylserine
CQ	Chloroquine
MOE	Molecular Operating Environment
RING	Really interesting new gene
RRP	Recurrent respiratory papillomatosis

## References

[1] Mattiuzzi C., Sanchis-Gomar F., Lippi G. (2019). Concise update on colorectal cancer epidemiology. Ann. Transl. Med..

[2] Giusti R.M., Shastri K.A., Cohen M.H., Keegan P., Pazdur R. (2007). FDA drug approval summary: Panitumumab (Vectibix). Oncologist.

[3] Ettrich T.J., Seufferlein T. (2014). Regorafenib. Small Molecules in Oncology.

[4] Graham J., Muhsin M., Kirkpatrick P. (2004). Cetuximab. Nat. Rev. Drug Discov..

[5] Van Cutsem E., Köhne C.-H., Hitre E., Zaluski J., Chang Chien C.-R., Makhson A., D’Haens G., Pintér T., Lim R., Bodoky G. (2009). Cetuximab and chemotherapy as initial treatment for metastatic colorectal cancer. N. Engl. J. Med..

[6] Oliner J.D., Saiki A.Y., Caenepeel S. (2016). The role of MDM2 amplification and overexpression in tumorigenesis. Cold Spring Harb. Perspect. Med..

[7] National Cancer Institute Clinical Trials Information: MDM2 Inhibitor. https://www.cancer.gov/about-cancer/treatment/clinical-trials/search/r?d=C162996&loc=0&pn=2&rl=2.

[8] Ding Q., Zhang Z., Liu J.-J., Jiang N., Zhang J., Ross T.M., Chu X.-J., Bartkovitz D., Podlaski F., Janson C. (2013). Discovery of RG7388, a potent and selective p53–MDM2 inhibitor in clinical development. J. Med. Chem..

[9] Vassilev L.T. (2004). Small-molecule antagonists of p53-MDM2 binding: Research tools and potential therapeutics. Cell Cycle.

[10] Li Y., Li X., Guo B. (2010). Chemopreventive agent 3,3′-diindolylmethane selectively induces proteasomal degradation of class I histone deacetylases. Cancer Res..

[11] Thomson C.A., Ho E., Strom M.B. (2016). Chemopreventive properties of 3,3′-diindolylmethane in breast cancer: Evidence from experimental and human studies. Nutr. Rev..

[12] Gamet-Payrastre L., Lumeau S., Gasc N., Cassar G., Rollin P., Tulliez J. (1998). Selective cytostatic and cytotoxic effects of glucosinolates hydrolysis products on human colon cancer cells in vitro. Anticancer Drugs.

[13] Kim E.J., Park S.Y., Shin H.K., Kwon D.Y., Surh Y.J., Park J.H. (2007). Activation of caspase-8 contributes to 3,3′-Diindolylmethane-induced apoptosis in colon cancer cells. J. Nutr..

[14] Choi H.J., Park J.H.Y. (2009). Induction of G1 and G2/M cell cycle arrests by the dietary compound 3, 3’-diindolylmethane in HT-29 human colon cancer cells. BMC Gastroenterol..

[15] Yun C., Dashwood W.-M., Li L., Yin T., Ulusan A.M., Shatzer K., Gao S., Ruan K.-H., Hu M. (2020). Acute changes in colonic PGE 2 levels as a biomarker of efficacy after treatment of the Pirc (F344/NTac-Apc am1137) rat with celecoxib. Inflamm. Res..

[16] Yun C., Dashwood W.-M., Kwong L.N., Gao S., Yin T., Ling Q., Singh R., Dashwood R.H., Hu M. (2018). Accurate quantification of PGE2 in the polyposis in rat colon (Pirc) model by surrogate analyte-based UPLC–MS/MS. J. Pharm. Biomed. Anal..

[17] Juven T., Barak Y., Zauberman A., George D., Oren M. (1993). Wild type p53 can mediate sequence-specific transactivation of an internal promoter within the mdm2 gene. Oncogene.

[18] Vogelstein B., Lane D., Levine A.J. (2000). Surfing the p53 network. Nature.

[19] Zhao Y., Yu H., Hu W. (2014). The regulation of MDM2 oncogene and its impact on human cancers. Acta Biochim. Et Biophys. Sin..

[20] Nag S., Qin J., Srivenugopal K.S., Wang M., Zhang R. (2013). The MDM2-p53 pathway revisited. J. Biomed. Res..

[21] Bohlman S., Manfredi J.J. (2014). p53-independent effects of Mdm2. Sub-Cell. Biochem..

[22] Ganguli G., Wasylyk B. (2003). p53-independent functions of MDM2. Mol. Cancer Res. MCR.

[23] Chou T., Martin N. (2007). CompuSyn Software for Drug Combinations and for General Dose-Effect Analysis, and User’s Guide.

[24] Rosen C.A., Bryson P.C. (2004). Indole-3-carbinol for recurrent respiratory papillomatosis: Long-term results. J. Voice.

[25] Anderton M.J., Manson M.M., Verschoyle R.D., Gescher A., Lamb J.H., Farmer P.B., Steward W.P., Williams M.L. (2004). Pharmacokinetics and tissue disposition of indole-3-carbinol and its acid condensation products after oral administration to mice. Clin. Cancer Res..

[26] Arneson D., Hurwitz A., McMahon L., Robaugh D. (1999). Presence of 3, 3′-Diindolylmethane in human plasma after oral administration of Indole-3-carbinol. Proc. Am. Assoc. Cancer Res..

[27] Thomson C.A., Chow H.H.S., Wertheim B.C., Roe D.J., Stopeck A., Maskarinec G., Altbach M., Chalasani P., Huang C., Strom M.B. (2017). A randomized, placebo-controlled trial of diindolylmethane for breast cancer biomarker modulation in patients taking tamoxifen. Breast Cancer Res. Treat..

[28] Dalessandri K.M., Firestone G.L., Fitch M.D., Bradlow H.L., Bjeldanes L.F. (2004). Pilot study: Effect of 3,3′-diindolylmethane supplements on urinary hormone metabolites in postmenopausal women with a history of early-stage breast cancer. Nutr. Cancer.

[29] Chang X., Tou J.C., Hong C., Kim H.A., Riby J.E., Firestone G.L., Bjeldanes L.F. (2005). 3,3′-Diindolylmethane inhibits angiogenesis and the growth of transplantable human breast carcinoma in athymic mice. Carcinogenesis.

[30] Bhatnagar N., Li X., Chen Y., Zhou X., Garrett S.H., Guo B. (2009). 3,3′-diindolylmethane enhances the efficacy of butyrate in colon cancer prevention through down-regulation of survivin. Cancer Prev. Res..

[31] Sepkovic D.W., Stein J., Carlisle A.D., Ksieski H.B., Auborn K., Bradlow H.L. (2009). Diindolylmethane inhibits cervical dysplasia, alters estrogen metabolism, and enhances immune response in the K14-HPV16 transgenic mouse model. Cancer Epidemiol. Biomark..

[32] Aronchik I., Kundu A., Quirit J.G., Firestone G.L. (2014). The antiproliferative response of indole-3-carbinol in human melanoma cells is triggered by an interaction with NEDD4-1 and disruption of wild-type PTEN degradation. Mol. Cancer Res..

[33] Lee Y.-R., Chen M., Lee J.D., Zhang J., Lin S.-Y., Fu T.-M., Chen H., Ishikawa T., Chiang S.-Y., Katon J. (2019). Reactivation of PTEN tumor suppressor for cancer treatment through inhibition of a MYC-WWP1 inhibitory pathway. Science.

[34] Wade M., Li Y.C., Wahl G.M. (2013). MDM2, MDMX and p53 in oncogenesis and cancer therapy. Nat. Rev. Cancer.

[35] Shangary S., Wang S. (2009). Small-molecule inhibitors of the MDM2-p53 protein-protein interaction to reactivate p53 function: A novel approach for cancer therapy. Annu. Rev. Pharmacol. Toxicol..

[36] Kojima K., Konopleva M., McQueen T., O’Brien S., Plunkett W., Andreeff M. (2006). Mdm2 inhibitor Nutlin-3a induces p53-mediated apoptosis by transcription-dependent and transcription-independent mechanisms and may overcome Atm-mediated resistance to fludarabine in chronic lymphocytic leukemia. Blood.

[37] Nag S., Zhang X., Srivenugopal K.S., Wang M.H., Wang W., Zhang R. (2014). Targeting MDM2-p53 interaction for cancer therapy: Are we there yet?. Curr. Med. Chem..

[38] Arena G., Riscal R., Linares L.K., Le Cam L. (2018). MDM2 controls gene expression independently of p53 in both normal and cancer cells. Cell Death Differ..

[39] Erba H.P., Becker P.S., Shami P.J., Grunwald M.R., Flesher D.L., Zhu M., Rasmussen E., Henary H.A., Anderson A.A., Wang E.S. (2019). Phase 1b study of the MDM2 inhibitor AMG 232 with or without trametinib in relapsed/refractory acute myeloid leukemia. Blood Adv..

[40] Khurana A., Shafer D.A. (2019). MDM2 antagonists as a novel treatment option for acute myeloid leukemia: Perspectives on the therapeutic potential of idasanutlin (RG7388). Oncotargets Ther..

[41] Honda R., Yasuda H. (1999). Association of p19ARF with Mdm2 inhibits ubiquitin ligase activity of Mdm2 for tumor suppressor p53. EMBO J..

[42] Gu J.J., Kawai H., Nie L.G., Kitao H., Wiederschain D., Jochemsen A.G., Parant J., Lozano G., Yuan Z.M. (2002). Mutual dependence of MDM2 and MDMX in their functional inactivation of p53. J. Biol. Chem..

[43] Okino S.T., Pookot D., Basak S., Dahiya R. (2009). Toxic and chemopreventive ligands preferentially activate distinct aryl hydrocarbon receptor pathways: Implications for cancer prevention. Cancer Prev. Res..

[44] Ohtake F., Fujii-Kuriyama Y., Kato S. (2009). AhR acts as an E3 ubiquitin ligase to modulate steroid receptor functions. Biochem. Pharmacol..

[45] (2018). Molecular Operating Environment (MOE), 2018.01.

[46] Leong H., Riby J.E., Firestone G.L., Bjeldanes L.F. (2004). Potent ligand-independent estrogen receptor activation by 3,3′-diindolylmethane is mediated by cross talk between the protein kinase A and mitogen-activated protein kinase signaling pathways. Mol Endocrinol..

