# Supplementary material for: Chemopreventive Agent 3,3′-Diindolylmethane Inhibits MDM2 in Colorectal Cancer Cells
Source: Int J Mol Sci. 2020 Jun 30;21(13):4642. doi: 10.3390/ijms21134642 (PMC7370074; doi:10.3390/ijms21134642)
Supplement: Supplementary file 1 [file ijms-21-04642-s001.pdf]

Supplementary Materials

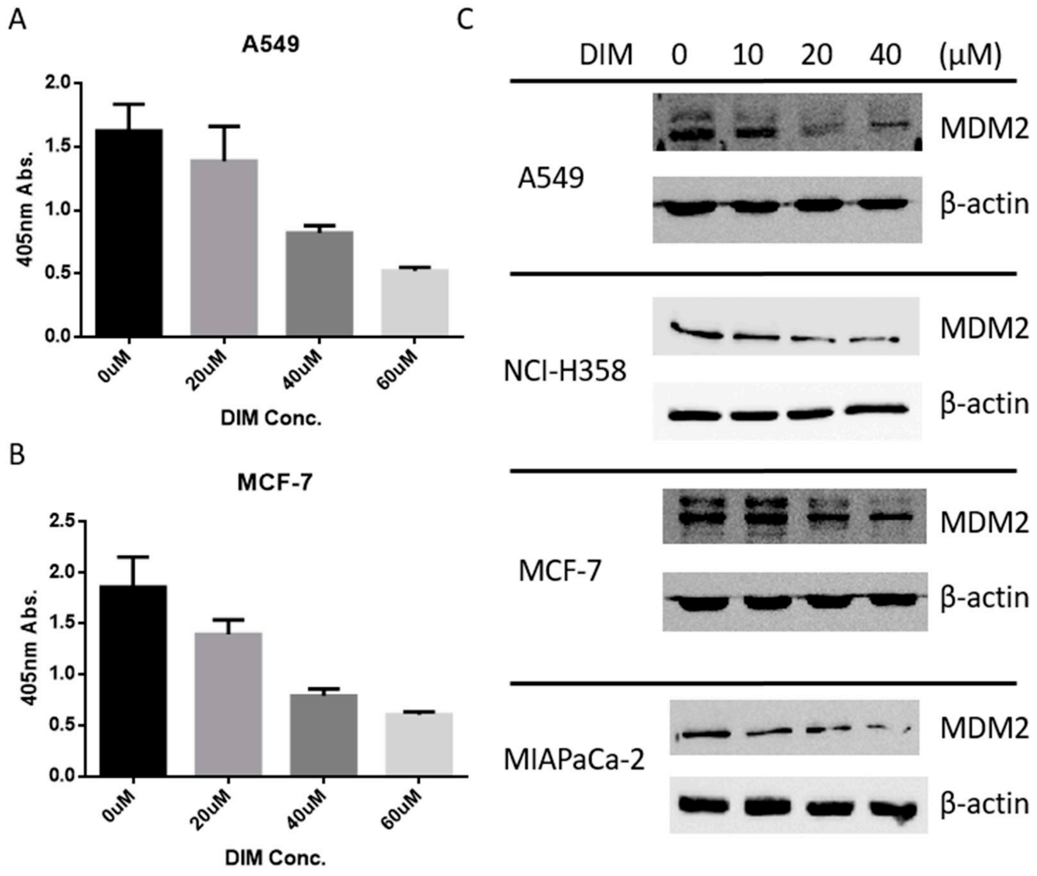

Figure S1. (A) and (B) DIM inhibits proliferation of various cancer cell lines. Cells were treated with various doses of DIM and cell proliferation was assessed by WST-1 assay. (C) DIM inhibits MDM2 protein in various cancer cell lines. Cells were incubated 24 hours with indicated concentrations of DIM (0 to 40 μM) before cell harvest and lysis. Cell lysates were analyzed by western blotting.

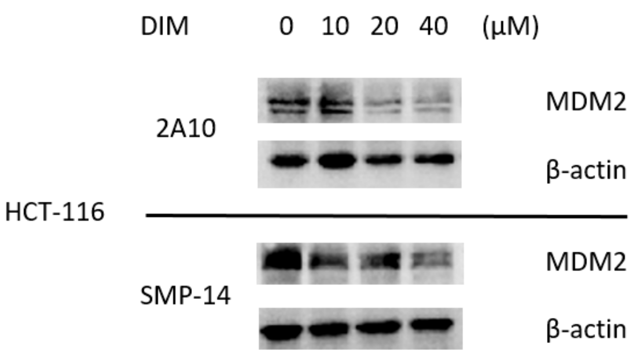

Figure S2. Clone 2A10 and SMP-14 antibodies were used to detect MDM2 protein in the same settings of DIM application towards HCT-116 cells. Both antibodies showed MDM2 protein decreased with increasing DIM concentrations.

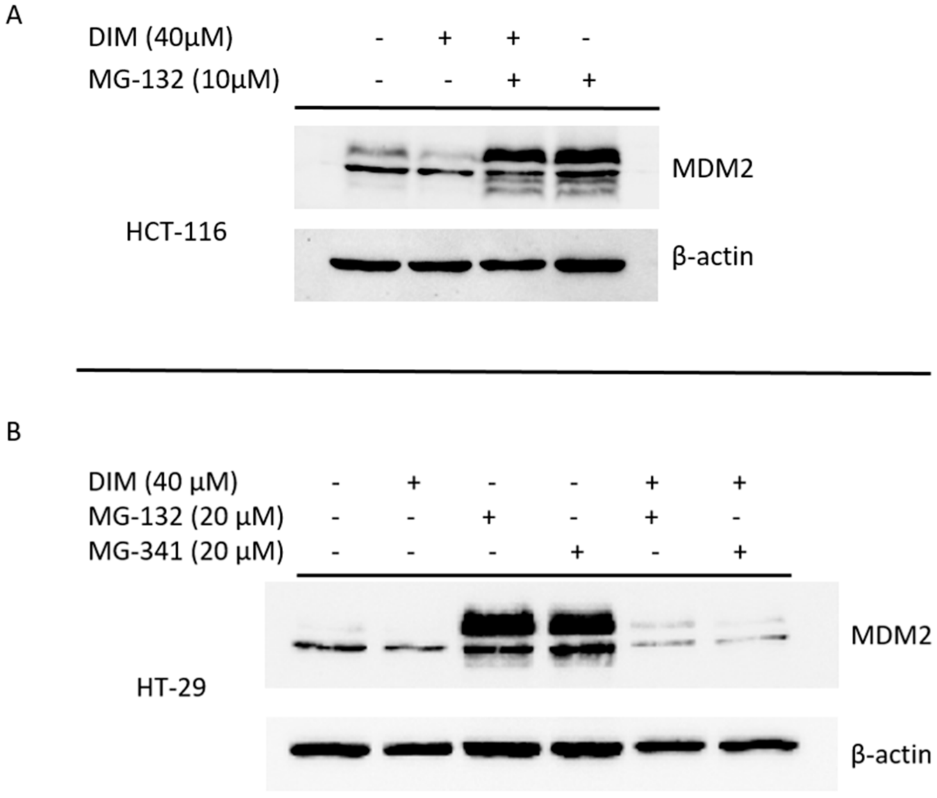

Figure S3. HCT-116 (A) and HT-29 (B) cells were treated with DIM alone or the combination of DIM and proteasome inhibitors at the indicated concentrations for 24h. Cell lysates were analyzed by Western blot.

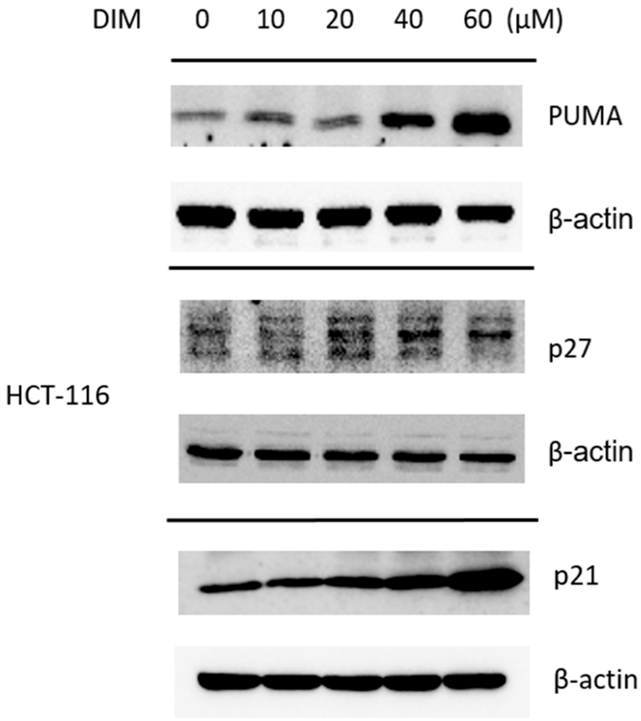

Figure S4. HCT-116 cells were treated with various doses of DIM for 24h. Cell lysates were analyzed by Western blot with antibodies against PUMA, p27, and p21 proteins.

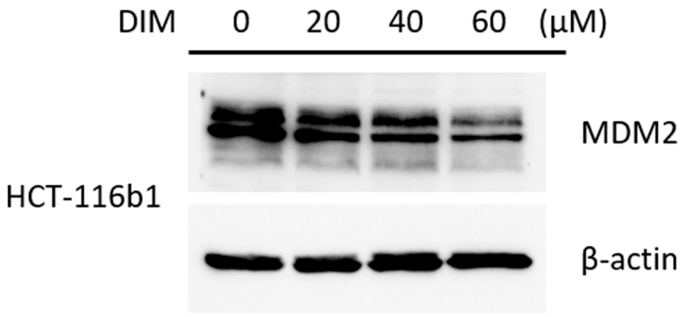

Figure S5. MDM2 overexpressing cell line, HCT-116b1, was treated with various concentrations of DIM for 24h. Cell lysates were analyzed by western blot.

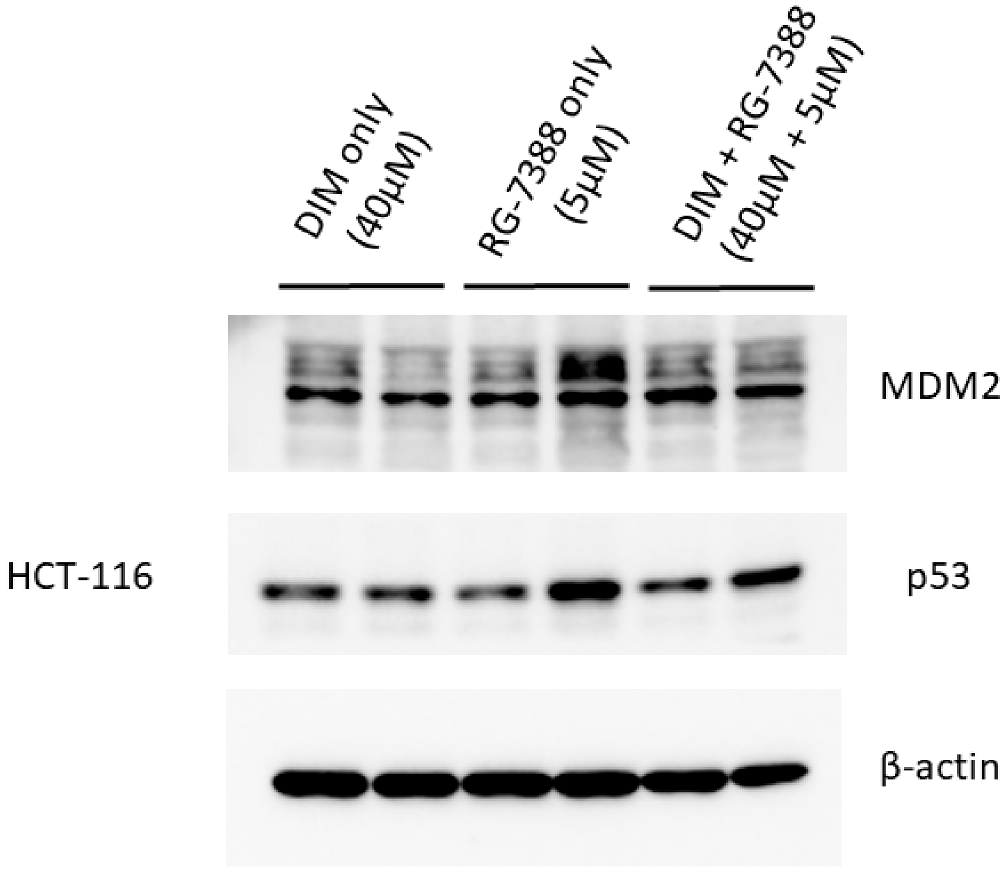

Figure S6. HCT-116 cells were treated with DIM, RG-7388, or the DIM/RG-7388 combination. Cell lysates were analyzed by western blot 24 hours after treatment with the indicated antibodies.
